# Enhancing the Cytotoxicity and Apoptotic Efficacy of Parasporin-2-Derived Variants (Mpp46Aa1) on Cancer Cell Lines

**DOI:** 10.3390/toxins16100415

**Published:** 2024-09-25

**Authors:** Juan S. Alarcón-Aldana, Lydia Visser, Nohora J. Rueda-Forero, Efraín H. Pinzón-Reyes, Paola Rondón-Villarreal, Miguel O. Suárez-Barrera

**Affiliations:** 1 Facultad de Ciencias Médicas y de la Salud, Instituto de Investigación MASIRA, Universidad de Santander, Bucaramanga 680002, Colombia; biomol.investigacion_1@udes.edu.co (J.S.A.-A.); juliana.forero@udes.edu.co (N.J.R.-F.); ehpinzon@udes.edu.co (E.H.P.-R.); diseno.molecular@udes.edu.co (P.R.-V.); 2 Department of Pathology and Medical Biology, University Medical Center Groningen, University of Groningen, 9700 AB Groningen, The Netherlands; l.visser@umcg.nl

**Keywords:** parasporin, site-directed mutagenesis, anti-cancer activity, in silico modeling

## Abstract

Parasporin PS2Aa1, recently renamed Mpp46Aa1, is an anti-cancer protein known for its selectivity against various human cancer cell lines. We genetically modified native PS2Aa1 to create a library of approximately 100 mutants. From this library, we selected promising mutants based on their half-maximal inhibitory concentration (IC_50_) and sequence variations. In this study, Variant 3–35, with the G257V substitution, demonstrated increased cytotoxicity and selectivity against the colon cancer cell line SW480. Conversely, Variant N65, featuring substitutions N92D, K175R, and S218G, yielded the most favorable results against the cancer cell lines SW-620, MOLT-4, and Jurkat. The caspase 3/7 and 9, Annexin V-Cy3 and 6-GFDA activities, and, most notably, mitochondrial membrane permeabilization assays confirmed the apoptotic marker elevation. These findings indicate that residues 92, 175, 218, and 257 may play a critical role in the cytotoxic activity and selectivity. We successfully obtained genetically improved variants with substitutions at these key amino acid positions. Additionally, we conducted molecular dynamic simulations to explore the potential interactions between PS2Aa1 and the CD59 GPI-anchored protein. The simulation results revealed that residues 57, 92, and 101 were consistently present, suggesting their possible significance in the interactions between parasporin and the CD59 protein.

## 1. Introduction

Cancer is currently one of the leading causes of death worldwide, with approximately 10 million reported deaths in 2020 [1]. Of the various types of cancer, colorectal cancer and leukemia are among the most common, accounting for around 2.5 million new cases and nearly 1.2 million deaths in 2020 [1,2]. Thus, it is imperative to explore new molecules and strategies for cancer treatment while minimizing side effects. One promising path is the use of parasporins, a group of proteins produced by *Bacillus thuringiensis* (*Bt*) [3,4,5,6]. To date, 19 parasporins have been identified and classified into six groups by the parasporin classification and nomenclature committee [7]. Among these, PS2Aa1 stands out for its remarkable cytotoxic activity against leukemia, colon cancer, and lung cancer when activated by proteinase K. Importantly, it has minimal or undetectable effects on non-cancerous cells [8,9,10].

PS2Aa1, known as Mpp46Aa1, is a β-pore-forming toxin (β-PFT), which is structurally related to aerolysin-like proteins [11]. Crystallographic studies revealed that Mpp46Aa1 comprised three distinct domains [9]. Domain I is the receptor-binding domain that interacts with a currently unknown putative receptor. Domains II and III are responsible for the assembly of oligomers and pore formation in the membrane. This process induces membrane permeabilization, alterations in cell morphology, organelle fragmentation, and, ultimately, cell death [8,9,11]. In terms of molecular events induced in sensitive cells, there is a strong indication that PS2Aa1 triggers apoptosis through the intrinsic pathway [3,6,10]. However, it remains necessary to investigate various important aspects, such as determining the main receptors of parasporins [8,10]. GPI-anchored proteins have been considered involved receptors [8,12,13,14]. Additionally, it is essential to consider the potential roles of mitochondrial permeabilization and oxidative stress in PS2Aa1’s mechanism of action, as these features align with the intrinsic apoptotic pathway [6,15].

Considering this knowledge gap and the possible function of crucial points in the structure of the protein, we exposed PS2Aa1 to genetic modification using site-directed mutagenesis supported by computational modeling to generate promising cancer treatment alternatives. In a previous study, we substituted key amino acids in loop 1 in domain I and generated a library of 71 possible mutants, from which we selected variant 3–35 (G257V) for its improved cytocidal activity against colon cancer cells compared to the native PS2Aa1 [16].

To understand the structure–function dynamics of PS2Aa1, we engineered variant N65 by employing a site-directed mutagenesis approach. In vitro assessments were conducted to evaluate its impact on cell viability, cytotoxicity, and apoptosis across various leukemia and colon cancer cell lines. Simultaneously, we employed modeling techniques to elucidate potential interactions involving the variant protein alongside comparisons with both native and variant 3–35 counterparts. This comprehensive study aims to shed light on the complex facets of parasporin’s functionality and its potential in cancer research.

## 2. Results

### 2.1. Construction of the Library of Mutants from PS2Aa1

Genomic DNA was isolated from the *Bt* 4R2 strain and amplified via PCR. A BLASTn analysis showed that the obtained ~1000 bp amplicon was >99% identical with PS2Aa1 (NCBI accession number: AB099515.1), which confirmed the obtention of the desired gene. The amplified gene encoding *ps2Aa1* was cloned into pET30a, and the restriction assay described previously confirmed the construct size [10]. From these amplifications, a library of 40 mutants was constructed in *E. coli* TOP 10. We then sequenced the mutants and selected candidates expressing non-silent mutations. According to MatGat, the identity and similarity of these sequences to ps2Aa1 reached 99.9%. Variants 0–15 and 3–35 (Table 1) were obtained from a previous study in our laboratory [16].

### 2.2. Protein Extraction

**Figure 1:**
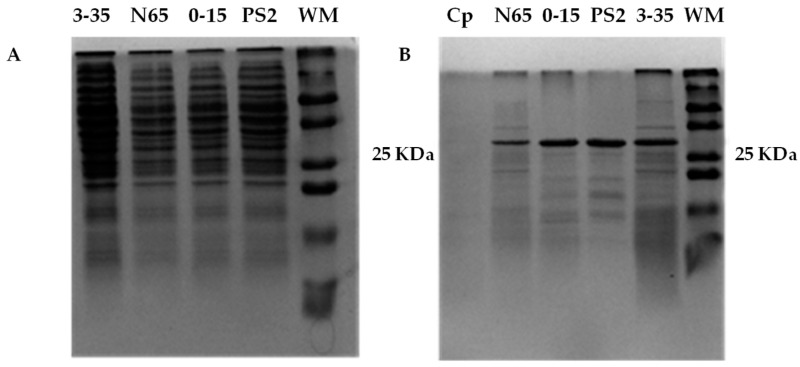
SDS-PAGE gel showing the 3–35, N65, 0–15, and PS2 (PS2Aa1) total protein extracts (**A**) and that treated with proteinase K (**B**). Cp: control of proteinase K without protein extract. WM: weight marker.

SDS-PAGE gels of the variants 0–15, 3–35, N65, and the recombinant PS2 were performed. The results showed multiple bands corresponding to the total extracted proteins (Figure 1A. These extracts were treated with proteinase K, cleaving the pro-protein to a size of approximately 30 kDa (Figure 1B).

### 2.3. Cytotoxicity of PS2Aa1 and Variants in Colon Cancer and Leukemia Cells

**Figure 2:**
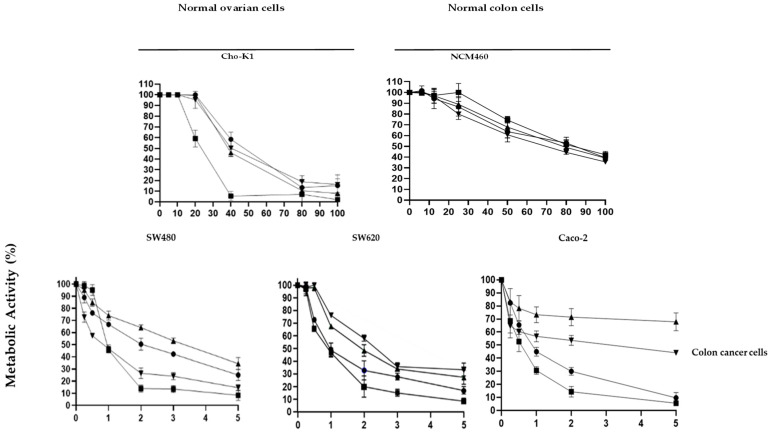

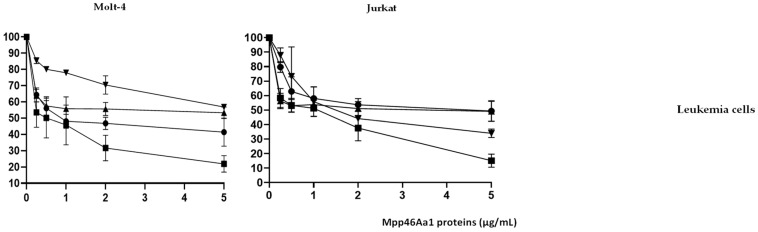
Cytotoxic activity of native PS2A1 and its variants after 48 h. Normal and cancer cell lines were treated with PS2 (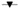) and variants 3–35 (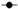), N65 (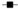), and 0–15 (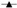) at different concentrations (from 0.5 to 5 µg/mL).

Cytotoxicity assays were performed using the Alamar blue assay (Figure 2 and Table 2). The cytocidal activity of the different proteins in normal cell lines NCM460 and CHO-K1 was observed at very high concentrations; CHO-K1, with IC_50_ values between 20 and 40 µg/mL, was more sensitive than NCM460, with IC_50_ values between 70 and 80 µg/mL (Table 2).

In SW480, both 3–35 and N65 were more cytotoxic than the native protein PS2Aa1, with IC_50_ values improving from 2.1 µg/mL to 0.9 and 1.2, respectively. In SW620 and MOLT-4, all three variants improved activity compared to the native protein, with N65 as the best-performing variant. In Jurkat, both N65 and 0–15 showed improvement, N65 being the best (Table 2). To determine the safety of these proteins’ use on a preclinical scale, selectivity values were determined for the activity of these proteins in cancer cells compared with their activity in the non-cancer cell line NCM460. As for a bioactive reference sample [17], the selectivity values above 10 were accepted for the case of a bioactive sample [17]. All three variants showed improved selectivity in all cell lines, with SW480 as the most promising selectivity index of 97.8 for 3–35 and variant N65 with a value of 131.3 for the leukemia cell MOLT-4; thus, these cell lines were selected for further experiments and toxic activity characterization (Table 3). The normal colon cell line NCM460 was used as a control.

### 2.4. PS2Aa1 Variants Induce Cell Death via Apoptotic Mechanisms in Colon Cancer and Leukemia Cells

**Figure 3:**
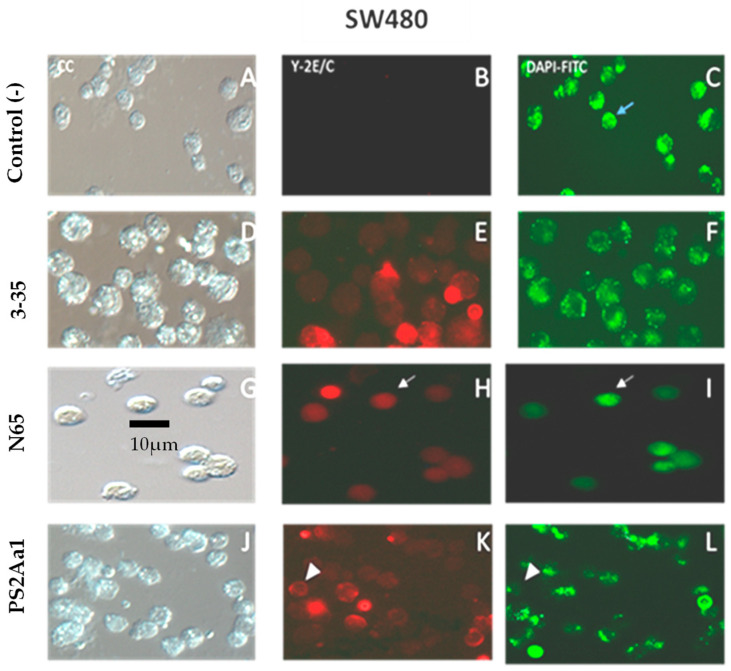
PS2Aa1 and its variants decrease cell capacity and increase phosphatidylserine exposure in SW480. Annexin V (Y–2E/C) (**B**,**E**,**H**,**K**) and DAPI–FITC (6–CFDA) (**C**,**F**,**I**,**L**) staining of the SW480 cell line treated with 5 µg/mL of toxins 3–35, N65, and PS2Aa1. Bright-field microscopy (CC) (**A**,**D**,**G**,**J**) was used to locate each cell. The blue arrow indicates a normal (viable) cell; the white arrows indicate a cell in early apoptosis, and the white arrowheads indicate a necrotic cell.

The PS2Aa1 and its variants have different IC_50_ values after 48 h for colon cancer and leukemia cell lines, ranging from 0.8 to 5 µg/mL (Table 2), so we standardized the concentration of all the following experiments to the maximum of these values (5 µg/mL). The reactions were performed for 24 h. To determine the possible mechanism of cell death in the target cells, a fluorescence assay with annexin V/Cy3 and 6-CFDA was performed on SW480 treated with PS2.r and variants 3–35 and N65. In this way, we aim to determine the translocation of phosphatidylserine to the outer cell membrane (red fluorescence, Annexin V/Cy3) and cell viability (green fluorescence, 6-CFDA). The increase in the percentage of cells with red fluorescence and the decrease in the percentage of cells with green fluorescence in all treatments indicated that parasporins 3–35, N65, and PS2.r could induce early apoptosis and cell death (Figure 3).

**Figure 4:**
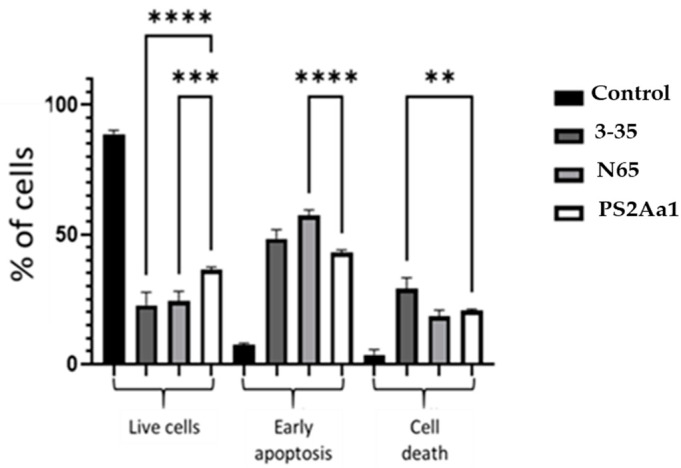
PS2Aa1 and its variants promote apoptosis in the SW480 cell line. The chart shows the percentage of live cells (Annexin V-Cy3-negative and 6-CFDA-positive), cells in early apoptosis (Annexin V-Cy3- and 6-CFDA-positive), and cells in the cell death phase (Annexin V-Cy3-positive and 6-CFDA-negative). Significant differences between control and treated cells were measured using 1-way ANOVA (** *p* < 0.01, *** *p* < 0.001, and **** *p* < 0.0001).

We analyzed 100 cells captured from three different fluorescence assays for each treatment. With the different treatments, the number of cells in apoptosis increased from 40% with the recombinant PS2Aa1 to 48 and 56% with 3–35 and N65, respectively (Figure 4). A statistical analysis to compare the variants with the recombinant protein was performed, and significant differences were observed with the N65 and 3–35 mutants in terms of cell viability and early apoptosis (Figure 4).

**Figure 5:**
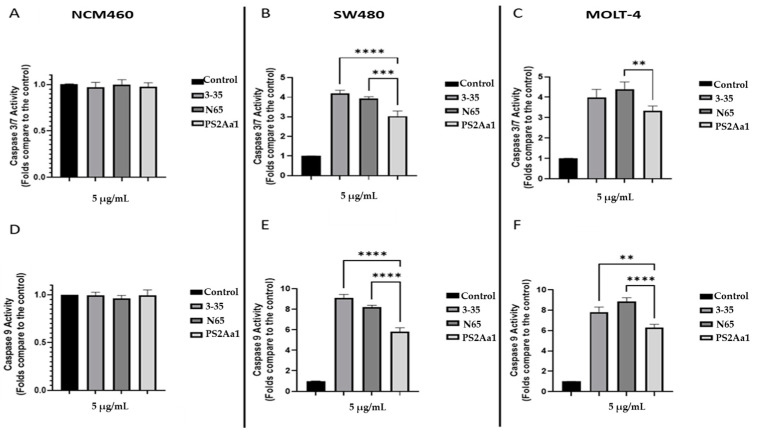
PS2Aa1 and variants increase the activation of caspases 3/7 and 9 in colorectal cancer and leukemia cells. NCM460 (**A**,**D**), W480 (**B**,**E**), and MOLT-4 (**C**,**F**) cells were treated with PS2.r proteins at 5 µg/mL for 24 h, and the level of caspases was measured using the Caspase-Glo^®^ 3/7 and Caspase-Glo^®^ 9 kit. Significant differences between control and treated cells were measured using 1-way ANOVA (** *p* < 0.01, *** *p* < 0.001, and **** *p* < 0.0001) compared to PS2Aa1 treatment.

To evaluate the possible induction of apoptosis by the recombinant PS2Aa1 and the variants N65 and 3–35, caspases 3/7 and 9 were measured in NCM460, SW480, and MOLT-4. Caspase 9 initiator caspase forms the intrinsic pathway, while caspase 3 is an executioner caspase common in intrinsic and extrinsic pathways. After 24 h of treatment, the proteins significantly increased the activity of caspases 3/7 and 9 in cancer cell lines. For SW480, the greatest increase in caspases 3/7 was with 3–35 treatment, increasing 4.1 times compared to the control without treatment. This result is similar to the increase in MOLT-4; in this cell line, the activity for those caspases was higher when we used the N65 treatment, being 4.4 times greater than the control (Figure 5B,C). Similarly, for caspase 9 activation, the best result in SW480 was with the variant 3–35, being 8.5 times the basal activity of the cells without treatment; in MOLT-4 cells, the treatment with N65 increased the activity to 8.6 times over the control (Figure 5E,F). These data correspond to the cytotoxicity assays since SW480 was more sensitive to 3–35, while MOLT-4 was more sensitive to N65 (Table 2).

**Figure 6:**
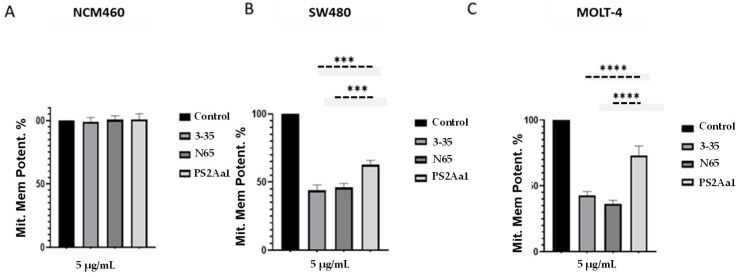
PS2Aa1 and variants induce mitochondrial membrane permeabilization in colon cancer and leukemia cells. Significant differences between control and treated cells were measured using 1-way ANOVA (*** *p* < 0.001, and **** *p* < 0.0001) compared to PS2Aa1 treatment.

To investigate whether there was mitochondrial damage in the cytocidal activity of the anti-cancer proteins, we measured the j-aggregates generated by the JC-1 reactive, which indicated mitochondrial membrane potential. Our results show a decrease in the mitochondrial membrane potential at 24 h of treatment with 5 µg/mL of the recombinant and variant toxins, indicating the permeabilization of the mitochondrial membrane. Additionally, there are significant differences in the permeabilization caused by PS2Aa1 in comparison with the effect of 3–35 and N65 mutants against SW480 and MOLT-4 (Figure 6B,C).

### 2.5. Molecular Dynamics of PS2Aa1 (Mpp46Aa1) Interaction with the Possible Main Receptor, GPI–CD59

As shown in Table 4, after three molecular dynamic simulation replicates, some PS2Aa1 residues made contact with CD59 or GPI for the longest simulation time (contact for more than 70% of the simulation time) and at a shorter average distance from the center of mass of the PS2Aa1 residue and the nearest GPI–CD59 complex residues (maintained interaction distances of less than 5 Å).

**Figure 7:**
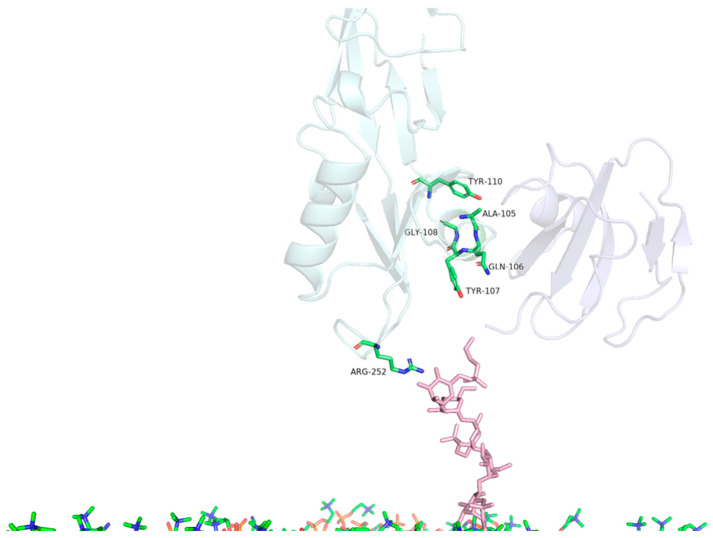
Identification of PS2Aa1 amino acids relevant for interaction with the GPI–CD59 complex. Highlighting the residues of PS2Aa1 ALA105, GLN106, TYR107, GLY108, TYR110 and ARG252.

The molecular dynamic results allow us to observe the involvement of a consistent group of PS2Aa1 protein amino acids that present a strong GPI–CD59 complex reaction; these amino acids are ALA105, GLN106, TYR107, GLY108, TYR110, and ARG252 (Table 4 and Table 5 and Figure 7).

**Figure 8:**
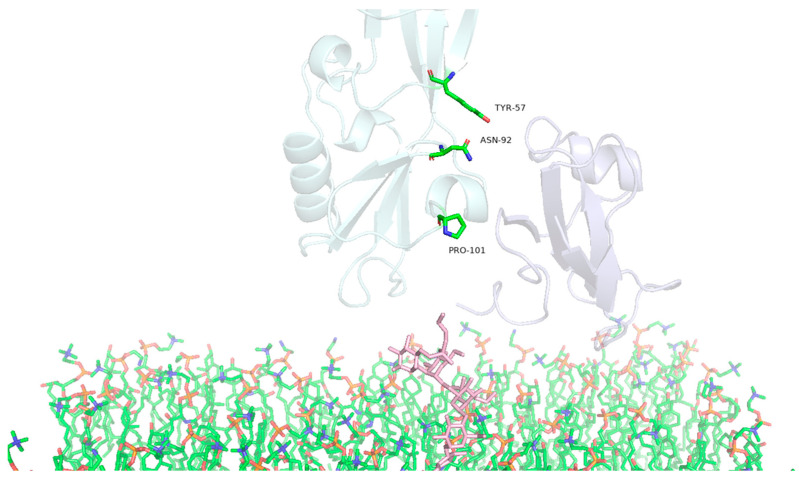
Identification of PS2Aa1 amino acids relevant with high prevalence for interaction with the GPI–CD59 complex. Highlighting the residues of PS2Aa1 TYR57, ASN92 and PRO101.

Another group of PS2Aa1 protein amino acids seems to be relevant in the interactions between the protein and the GPI–CD59 complex since they are found in two of the three replicates of the computational simulations; these amino acids are TYR57, ASN92, and PRO101 (Table 4, Figure 8).

**Figure 9:**
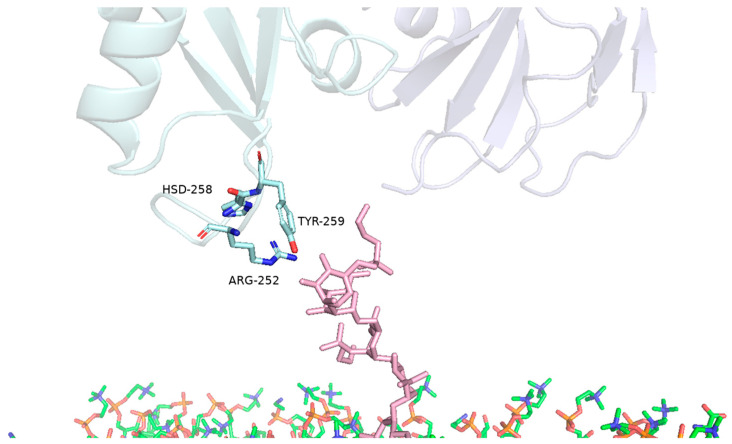
Identification of PS2Aa1 amino acids relevant for interaction with the glycan core of GPI–CD59 complex. Highlighting the residues of PS2Aa1 ARG252, HSD258 and TYR259.

As can be seen, some amino acids of the parasporin show interaction with the GPI of the complex; in this interaction, the amino acid ARG252 is highly relevant, participating actively in all computational simulations, while two additional amino acids, HIS258 and TYR259, participate in the simulations for an important period with 59% and 56% contacts, respectively (Figure 9).

## 3. Discussion

Since their discovery about 20 years ago, parasporins have attracted attention because of their selective toxic effects on various types of cancer cells [17]. As a result, they have been proposed as a therapeutic alternative. It is important to understand how these proteins function, what they trigger in target cells, and why they typically affect only these cell types. PS2Aa1 [Mpp46Aa1) is one of the parasporins with the broadest anti-cancer spectrum [7]. Therefore, the aim of this study was to investigate the mechanism of action by characterizing the variants obtained via site-directed mutagenesis.

Using PS2Aa1 as a template and based on preliminary studies showing an anti-cancer effect of peptides from loop 1 of PS2Aa1 on SW480 and SW620 cell lines [18], mutants were generated with random substitutions in amino acids G254, P255, G256, and G257. In addition, a variant obtained in a previous study with substitutions at sites N92D, K175R, and S218G—the N65 mutant—was also included. The variant subset selected with different activity levels enabled an exploration of the role of different specific protein regions of P2Aa1 [16,19]. Based on the IC50 and toxin selectivity index, the best variants, 3–35 and N65, were selected for characterization.

Regarding the different parasporins’ activity, variant 0–15 showed notably higher activity against CHOK1 compared with the other variants in all cell lines, except for NCM-460 and Caco-2, where its function was maintained and decreased 30-fold, respectively, compared with the native protein. On the other hand, the variants that improved cytotoxicity against all the cancer cell lines evaluated over the native protein were variants 3–35, whose substitution is found in loop 1 of domain 1 at the beginning of the β12-layer, and variant N65, which has three substitutions in each of the protein domains, starting with β-layers 3, 8, and 11. The substitution (N92D of N65 and G257V of 3–35) in domain 1 could increase parasporin’s affinity for important parasporin activity receptors, such as GPI-ap [12,13]. The second substitution (K175R in N65) is located in domain 2, which is mainly involved in pore formation and could change the pore’s nature to promote greater ion or protein efflux [8]; the third substitution (S218G EN N65) in domain 3, involved in oligomerization, could, in turn, increase the number of pores formed, as well as the percentage of dead cells [10,11,20] (Figure 7). The case of N65 is interesting because it is possible that only one of its substitutions causes the genetic enhancement, and the others are not involved in this process; on the other hand, it is also possible that two or more substitutions help enhance the selective effect against cancer cell lines; this variant requires further studies.

We must accurately identify these critical points, not only to determine that they cannot interact with the protein but also to see whether changing more than one residue can provide better results. Similarly, site-directed mutagenesis can trigger activity in a different type of target cell, as previously demonstrated using this breeding strategy with Cry3A, another protein produced by Bt. Through changes in loops 3 and 4 of domain I, which are responsible for the oligomerization of these proteins, it began to show activity in Diabotrica virgifera, decreasing from an LC50 of >100 µg/mL to 65 µg/mL [21]. Proteins with increased activity can be obtained using a rational design technique, as was the case with Cry2a. This involved first eliminating aspartic acid 42 (D42), which resulted in a two- to three-fold increase in cytotoxicity, then replacing lysine 63 with phenylalanine (K63F), which improved activity by about 50%, and finally replacing lysine 64 with proline (K64P), which increased toxin activity against three pests of the order Lepidoptera by about 40%. The overall improvement was 4.1- to 6.6-fold compared to the native protein [22]. Therefore, the enhanced variants treated in this project could have a broader spectrum of activity against different types of cancer cells. It should not be forgotten that this technique could be used to generate more and better mutants and identify the critical points that improved activity.

Regarding the characterization of the proteins’ anti-cancer activity, both the native protein and its variants increased phosphatidylserine exposure in SW480, consistent with reports in the literature on the apoptotic activity of PS2Aa1 [10,23]. There is evidence of apoptosis via the mitochondrial pathway, such as mitochondrial fragmentation and the activation of caspases 3/7 and 9 with up to a six-fold increase when liver cancer cells (HEPG2) were treated with PS2Aa1a at a concentration of 2 µg/mL for 24 h [10].

The action mechanism of these proteins involves the intrinsic pathway of apoptosis, observed from an increase in cleavage of caspases 3/7 and 9; this has been reported previously [15,24]. Other proteins homologous to PS2Aa1 have also shown apoptotic activity via the intrinsic pathway in their target cells. One example is the α-toxin of *Staphylococcus aureus*, a pore-forming protein active in the Jurkat cell line through apoptosis. It increases the efflux of cytochrome C from mitochondria and the activity of caspases 3 and 9 [25]. Other parasporins have also been reported to promote this apoptotic pathway in the cells they act upon, such as the 29-kDa protein isolated from Bt in India, which induces caspases 3 and 9 cleavage in the uterine cancer cell line HeLa [3]; activation of these caspases by parasporin 4 (PS4Aa1/MPP45Ba1) was also found in the MOLT-4 cell line [26].

This work also aimed to investigate whether there was an oxidative response in cell lines affected by recombinant parasporin and its variants, as various previous studies have shown evidence of the accumulation of ROS by different types of pore-forming proteins. Analogs to PS2Aa1 include α-toxins, aerolysin, and insecticidal Cry proteins [27,28,29]. Parasporin 3, also known as Cry41Aa, was found not to increase hydrogen peroxide (H_2_O_2_) in HepG2 [30]. Along these lines, a recent paper examined two parasporal proteins, 26 and 30 kDa, active against MCF-7 (breast cancer). However, the toxin did not result in a statistically significant increase in ROS levels after 48 h of treatment in MCF-7 cells [6]. Our results suggest that treatment with the selected variants did not have an effect on ROS levels in cancer cell lines.

Characterization of the anti-cancer activity of the investigated parasporins was performed to propose a possibly more structured and complete mechanism of action based on the reports previously published in the literature. PS2Aa1 may behave analogously to aerolysins, which have four domains. Domain I is involved in binding to oligosaccharides and associates with a β-sheet-rich lobe composed of the other three domains; domain II is responsible for binding to the major receptor, the glycan center of GPI; and domains III and IV are involved in oligomerization and pore formation. In this order and conception, domain II of this toxin would bind to the glycan center of GPI; later, oligomerization would occur through interactions between the domains III of seven aerolysin monomers and generate an inverted fungal structure; finally, the domains III and IV would form a β-barrel and generate the lytic pore [8,11,31].

**Figure 10:**
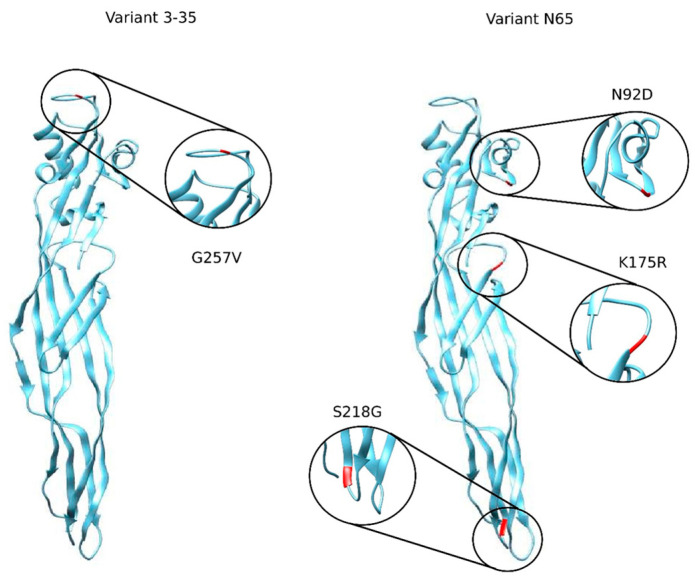
Three-dimensional modeling of variants 3–35 and N65. The specific location of each of the mutations present in the two most promising variants is indicated. The modeling was carried out using the Discovery program.

Subsequently, Abe et al. proposed a model consistent with this proposed mechanism for aerolysin [32]. According to this model, domain I of PS2Aa1 would recognize the putative receptor, and the glycan center of GPI might enhance this interaction to promote oligomerization by domain III [8]. At this point, the reverse fungal structure would potentially form, and the lytic pore would be generated with the β-sheets of the II domain, resulting in protein and ion efflux (Figure 10). This would generate intrinsic lethal stimuli, of which the accumulation of intracellular ROS is not one. This would potentially trigger BH-3 protein activation, which, in turn, could promote the activation of the BAX and BAK enzymes, which are responsible for the mitochondrial membrane permeability demonstrated in the present study, followed by cleavage of SMAC and cytochrome c in the cytoplasm; the SMAC protein would inhibit XIAP activation, a protein that promotes caspases 3/7 and 9 activation, thus preventing the apoptotic cascade. On the other hand, cytochrome C would activate the apoptosome and cause caspase 3/7 and 9 activation, as shown in this project (Figure 6). It should be noted that activation with proteinase K also affected the rest of the proteins produced by the bacteria, degrading them until the different bands were barely visible, leaving a predominant band corresponding to the activated protein (Figure 1B).

## 4. Conclusions

The study of Mpp46Aa1 (formerly Parasporin PS2Aa1) and its genetically modified variants has highlighted significant advancements in its anti-cancer properties. Variant 3–35, characterized by the G257V substitution, demonstrated enhanced cytotoxicity and selectivity against SW480 colon cancer cells. Conversely, variant N65, featuring substitutions N92D, K175R, and S218G, showed promising efficacy against SW-620, MOLT-4, and Jurkat cancer cell lines. Molecular assays confirmed elevated apoptotic markers, suggesting the critical roles of residues 92, 175, 218, and 257 in these activities. Molecular dynamics simulations further indicated potential interactions between Mpp46Aa1 and CD59, with residues 57, 92, and 101 identified as potentially significant. These findings underscore the successful development of improved variants and provide insights into the molecular mechanisms underlying the selective cytotoxicity of Mpp46Aa1 against various cancer cell types. Future research could further elucidate these interactions to optimize therapeutic applications of PS2Aa1 in cancer treatment.

## 5. Materials and Methods

### 5.1. Bacterial Strains and Culture Condition

*Bt* serovar Dakota strain 4R2, harboring ps2Aa1, was obtained from *Bacillus* Genetic Stock Centre (Ohio State University, Columbus, OH, USA) and cultured at 30 °C in Luria–Bertani (LB) broth. *E. coli* strain DE3BL21 and Bt mutant strain BMB171 with construct pET30a + ps2Aa1 were cultured in LB broth at 37 and 30 °C with agitation at 200 rpm, respectively.

### 5.2. Cell Culture Conditions

Human T lymphocyte leukemia cells (Jurkat), human T lymphoblast leukemia cells (MOLT-4), human colon adenocarcinoma cells (SW480, SW620, and Caco-2), human colon mucosal epithelial cells (NCM460; non-cancerous cells), and Chinese hamster ovary (CHO-K1; non-cancerous cells) cell lines were obtained from the American Type Culture Collection (ATCC, Manassas, VA, USA). SW480, SW620, Caco-2, NCM460, and CHO-K1 cell lines were maintained in Dulbecco’s Modified Eagle’s Medium—high glucose (DMEM, Thermo Fisher, Lenexa, KS, USA). MOLT-4 and Jurkat cell lines were grown in Roswell Park Memorial Institute Medium (RPMI 1640). Both culture media were supplemented with 10% fetal bovine serum (FBS, Thermo Fisher, Lenexa, KS, USA), 100 U/mL penicillin, 100 µg/mL streptomycin (Gibco, Thermo Fisher, Lenexa, KS, USA), 2 mM L-glutamine, 1 mM sodium pyruvate (Gibco), and 1% MEM Non-Essential Amino Acid Solution (Gibco). All cultures were incubated in a humidified incubator at 37 °C under a 5% CO_2_ atmosphere.

### 5.3. Generation of Parasporin Mutants from Site-Directed Mutagenesis

*ps2Aa1* from Bt 4r2 was amplified using a PCR protocol with the primers and amplification conditions established previously [10]. Mutants were selected according to their cytotoxicity. For the verification of the mutants, we used the procedure previously reported [16]. Briefly, the mutagenesis reaction was employed to transform TOP10F cells; 80 µL of the transformed cells was inoculated in LB plates with 25 µg/mL of kanamycin and X-gal overnight at 37 °C. Then, 40 white colonies were selected and cultured in LB broth in agitation at 37 °C overnight. The Wizard Plus SV Minipreps DNA purification system (Promega^®^, Madison, WA, USA) was used to extract plasmid DNA according to the manufacturer’s instructions. Sequencing of the desired fragments using universal T7 primers was performed using Macrogen^®^ (Seoul, Republic of Korea). The sequences were analyzed with Bio-Edit Software V 7.7 to identify the specific substitutions of each selected variant; finally, we used this DNA plasmid to electroporate the *Bt* Cry negative strain BMB171 with two electroshocks of 1.5 kV during 4.5 ms.

### 5.4. Extraction and Activation of Parasporins

Recombinant BMB 171-carrying ps2Aa1 and variants 3–35, N65, and 0–15 were cultured for 5 days at 30 °C with constant agitation. The cultures were centrifuged at 10,000 rpm for 10 min, and the resulting pellet was washed twice with water. The pellet containing parasporal crystals was dissolved in 2 mL of calcium carbonate solution containing 56 mM Na_2_CO_3_ (pH: 11.4) and 11 mM dithiothreitol (DTT) at 37 °C for 1.5 h. The lysed material was then centrifuged at 14,000 rpm for 5 min, and the supernatant passed through a 0.22 µm membrane filter (Millipore Merck, Rahway, NJ, USA). Finally, the pH of the filtrate was adjusted to 8 [10] with 1 M Tris–HCl (pH 5). Proteins were activated with proteinase K at a final concentration of 180 μg/mL for 1 h at 37 °C. Finally, proteinase K inactivation was performed using phenylmethylsulfonylfluoride at a final concentration of 1 mM, and SDS-PAGE gels were run to monitor protein activation [10].

### 5.5. Cytotoxicity Assay and Selectivity Calculation

For the determination of the cytotoxic activity of recombinant and variant proteins Sigma-Aldrich’s (St. Louis, MO, USA) cell viability reagent Alamar Blue HS (high sensitivity) was used. Briefly, cell concentration was determined with an automated cell counter (Countess II FL; Thermo Fisher, Lenexa, KS, USA), and 8000 cells (for SW480, SW620, Cho-k1, and NCM460) and 20,000 cells (for Jurkat and MOLT-4) were seeded into 96-well plates and incubated in DMEM or RPMI medium (Gibco) at 37 °C with 5% CO_2_. Moreover, 0.025, 0.5, 1, 2, 3, and 5 µg/mL parasporin variants were added, and the cells were cultured for 48 h. The final concentration of solubilization buffer was 10% and maintained throughout all experiments. Finally, the IC_50_ was determined by adding 10 µL of Alamar Blue to each well, incubating the samples for 5 h, and measuring fluorescence (Ex 560 nm and Em 590 nm). Each experiment was performed in triplicate. Proteinase K without protein extract was used as a control. The results are expressed in terms of metabolic activity using the following formula:

$$\%\;\mathrm{Metabolic}\;\mathrm{activity}=\frac{Absorbance\;of\;treated\;cells}{Absorbance\;of\;untreated\;cells}*100$$

### 5.6. Apoptosis Detection via Annexin V-Cy3/6-DFDA Assay

The apoptosis detection kit Annexin V-CY3 from Sigma-Aldrich (St. Louis, MO, USA) was used according to the manufacturer’s instructions. NCM460, SW480, and MOLT-4 cells (1 × 10^6^ cells/mL) were treated with 5 µg/mL parasporin and incubated at 37 °C in 5% CO_2_ atmosphere for 24 h. Cells were washed twice with PBS, trypsinized (for NCM460 and SW480), and resuspended in PBS. A total of 50 μL of cell suspension was then plated onto PolyPrep poly-L-lysine coated slides, washed, and labeled with Annexin V-Cy3 + 6-CFDA solution for 10 min. Slides were washed, rehydrated, and covered. Fluorescence was measured with an EVOS M7000 digital inverted fluorescence microscope (ThermoFisher Scientific, Waltham, MA, USA). The red fluorescence of Annexin V-Cy3 (Ex = 555 nm, Em = 569 nm) and the green fluorescence of 6-CFDA (Ex = 492 nm, Em = 517 nm) were visualized using the DAPI–FITC–Texas Red emission filters. The experiment was performed in triplicate.

### 5.7. Caspases 3/7 and 9 Assay

A luminescence assay was carried out using the Caspase-Glo 3/7 and Caspase-Glo 9 kits (Promega, Madison, WI, USA). Briefly, the cells were incubated with 5 µg/mL of parasporin variants at 37 °C, 5% CO_2_ for 24 h. Then, 100 μL of Caspase-Glo 3/7 and 9 reagents were added to the wells according to the manufacturer’s instructions. One hour later, the luminescence was measured using a Varioskan LUX Multimode Microplate Reader (Thermo Scientific, Waltham, MA, USA). The results are expressed in terms of fold change compared to the control. All experiments were performed in triplicate.

### 5.8. Determination of Mitochondrial Permeability Potential

To determine mitochondrial membrane potential, tetraethylbenzimidazolylcarbocyanine-6,6′-tetrachloro-1,1′,3,3′ iodide (JC-1) (Abcam PLC, Cambridge, UK) was used. JC-1 forms red aggregates in healthy cell mitochondria with high mitochondrial membrane potential, while it is green as a monomer in apoptotic cells and indicates a low mitochondrial membrane potential [33,34]. Cells were incubated in 96-well plates (20,000 cells/well) with 20 µM JC-1 dye for 15 min at 37 °C in 5% CO_2_ and washed twice with PBS. The results were measured in a Varioskan LUX multimode microplate reader at excitation/emission wavelengths of 475/530 nm (red).

### 5.9. Molecular Dynamic Analysis

Molecular dynamic simulations were used to study the possible implications of parasporin PS2Aa1’s interaction with GPI-anchored receptors in colon cancer cells. The modeled system was composed of a cancer membrane bilayer, a GPI attached to the lipid membrane, the GPI-anchored protein CD59, and the native *PS2Aa1*. The CD59 protein was selected as a possible interacting receptor of these proteins, considering that it is an overexpressed protein in colorectal cancer cells compared to normal colorectal cells, according to the information reported in the human protein atlas [35,36].

The 3D structures of native PS2Aa1 and CD59 were obtained from the Protein Data Bank [37] under the identifiers 2ZTB and 1CDR, respectively (Figure 1). The initial system structures were built with the CHARMM22.0 online software [38], using the lipid composition of a cancerous eukaryotic cell given in a study by Marco Klähn and Martin Zacharias [39]. The water thickness was set to 80 Å, and K^+^ and Cl^−^ were added to neutralize the system.

**Figure 11:**
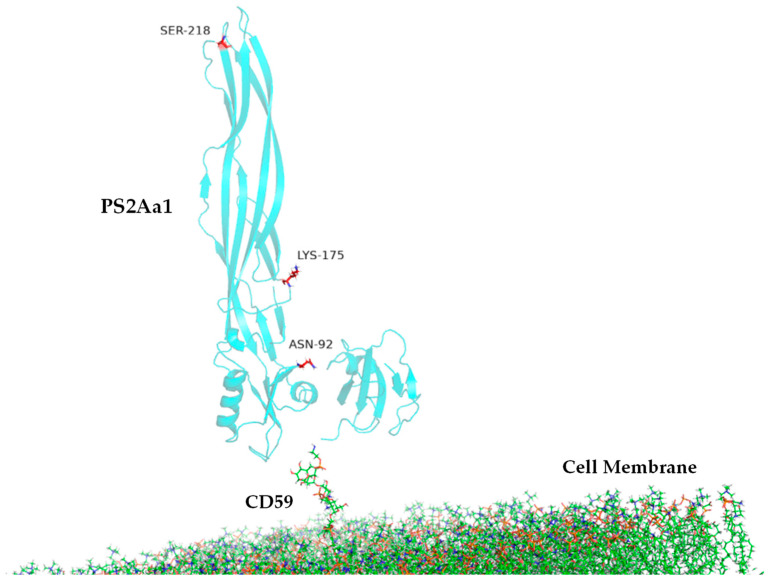
System PS2Aa1 (MPP46Aa1) CD59–GPI complexes on membrane.

The modeled system PS2Aa1–CD59–GPI attached to the membrane was first minimized and equilibrated using Amber18 software. The Amber ff19SB force field for proteins was implemented for all simulations (Figure 11).

Complexes were first minimized for 5000 (steepest descent) and 10,000 (conjugate gradient), followed by heating for 1 ns and then in an NVT ensemble (canonical ensemble) to 300 K. This included solvation with TIP3P water molecules, and Na^+^ and Cl^−^ ions were added to ensure the system neutrality at an ion concentration of 150 mM. Simulations were performed in triplicate; for each, an 8 Å cutoff was used for unbounded Coulombic and Lennard–Jones interactions and for periodic boundary conditions with a particle-mesh Ewald treatment for long-range Coulombic interactions. The SHAKE algorithm employed a time step of 2 fs, and the production steps were 100 ns for the systems [16].

### 5.10. Statistical Analysis

The data were analyzed using one-way ANOVA (analysis of variance) in GraphPad Prism 10 software. Differences between each treatment and the control were determined via the Dunnett test, with a statistical significance of * *p* ≤ 0.05, ** *p* ≤ 0.01, or *** *p* ≤ 0.001, with 95% CIs.

## Figures and Tables

**Table 1:** Mutations in the variants obtained through site-specific mutagenesis selected according to their cytotoxic activity.

Nomenclature	Type/Location of the Mutation	Notation of the Mutation
PS2Aa1	PS2Aa1 (PDB 2ZTB)	-
0–15	SUS/256	G256A
3–35	SUS/257	G257V
N65	SUS/92,175,218	N92D, K175R, S218G

**Table 2:** Cytotoxicity assays with Alamar Blue show the half-maximal inhibitory concentration (IC_50_) of the toxins against all treated cell lines.

Parasporin/Cell Line	IC_50_ of Parasporins in µg/mL (95% CI)						
	SW480	SW620	CaCO-2	MOLT-4	Jurkat	CHOK1	NCM460
0–15	2.9 (2.2–3.4)	2 (1.6–2.5)	73.32 (11.6–122.4)	1.7 (0.8–3.6)	1.2 (0.6–2.6)	20.2 (20–20.3)	77.7 (71.6–8)
3–35	0.9 (0.6–1.1)	1.2 (0.9–1.5)	0.88 (0.81–0.9)	1.0 (0.6–1.8)	1.9 (1.2–3.2)	39.9 (39.8–4)	78.8 (70.5–89.6)
N65	1.2 (1–1.3)	1.0 (0.9–1.2)	0.51 (0.427–0.6)	0.6 (0.4–0.9)	0.7 (0.5–1)	40 (39.8–40.2)	84.2 (79.2–9)
PS2Aa1	2.1 (1.5–2.8)	2.3 (1.7–2.9)	2.57 (1.84–4.1)	4.7 (3.6–6.5)	1.6 (1.2–2.2)	40.1 (40–40.3)	67.7 (63.6–72.1)

**Table 3:** Selectivity index for each of the toxins. The values were found with the IC_50_ of the toxins in each cancer cell line to the data obtained in the non-cancer cell line (NCM460).

Parasporin/Cell Lines	SW480	SW620	CaCo-2	MOLT-4	Jurkat
0–15	27.0	38.2	1.1	45.7	63.7
N65	67.4	77.1	165.1	131.3	107.9
3–35	97.8	72.5	89.5	78.7	43.4
PS2Aa1	31.9	29.5	26.3	14.2	42.3

**Table 4:** The residues wherein PS2Aa1 made contact with GPI–CD59 at 70% frequency are shown for each independent molecular dynamic (MD) run.

MD	Contact Residues (>70%)	
	PS2Aa1 Residues—CD59	PS2Aa1 Residues—GPI
Replicate 1	ALA105, GLN106, TYR107, GLY108, TYR110	ARG252
Replicate 2	TYR57, ASN92, PRO101, ALA105, GLN106, TYR107, GLY108, TYR110	ARG252
Replicate 3	TYR57, ASN92, PRO101, ALA105, GLN106, TYR107, GLY108, TYR110	ARG252

**Table 5:** PS2Aa1 residue distance relevant to interaction with CD59.

Comparison	PS2Aa1 Residues—CD59
ALA105	5.6 Å
GLN106	5.7 Å
TYR107	3.6 Å
GLY108	4.8 Å
TYR110	3.8 Å
TYR57	5.4 Å
ASN92	5.6 Å
PRO101	3.6 Å

## Data Availability

The corresponding author will make the raw data supporting the conclusions of this article available upon request.

## References

[1] Sung H., Ferlay J., Siegel R.L., Laversanne M., Soerjomataram I., Jemal A., Bray F. (2021). Global Cancer Statistics 2020: GLOBOCAN Estimates of Incidence and Mortality Worldwide for 36 Cancers in 185 Countries. CA Cancer J. Clin..

[2] Debela D.T., Muzazu S.G.Y., Heraro K.D., Ndalama M.T., Mesele B.W., Haile D.C., Kitui S.K., Manyazewal T. (2021). New Approaches and Procedures for Cancer Treatment: Current Perspectives. SAGE Open Med..

[3] Chubicka T., Girija D., Deepa K., Salini S., Meera N., Raghavamenon A.C., Divya M.K., Babu T.D. (2018). A Parasporin from Bacillus Thuringiensis Native to Peninsular India Induces Apoptosis in Cancer Cells through Intrinsic Pathway. J. Biosci..

[4] Palma L., Munoz D., Berry C., Murillo J., Caballero P. (2014). Bacillus Thuringiensis Toxins: An Overview of Their Biocidal Activity. Toxins.

[5] Okassov A., Nersesyan A., Kitada S., Ilin A. (2015). Parasporins as New Natural Anticancer Agents: A Review. J. BUON.

[6] Borin D.B., Castrejón-Arroyo K., Cruz-Nolasco A., Peña-Rico M., Rorato M.S., Santos R.C.V., de Baco L.S., Pérez-Picaso L., Camacho L., Navarro-Mtz A.K. (2021). Parasporin A13-2 of Bacillus Thuringiensis Isolates from the Papaloapan Region (Mexico) Induce a Cytotoxic Effect by Late Apoptosis against Breast Cancer Cells. Toxins.

[7] Akiba T., Okumura S. (2017). Parasporins 1 and 2: Their Structure and Activity. J. Invertebr. Pathol..

[8] Abe Y., Inoue H., Ashida H., Maeda Y., Kinoshita T., Kitada S. (2017). Glycan Region of GPI Anchored-Protein Is Required for Cytocidal Oligomerization of an Anticancer Parasporin-2, Cry46Aa1 Protein, from Bacillus Thuringiensis Strain A1547. J. Invertebr. Pathol..

[9] Akiba T., Abe Y., Kitada S., Kusaka Y., Ito A., Ichimatsu T., Katayama H., Akao T., Higuchi K., Mizuki E. (2009). Crystal Structure of the Parasporin-2 Bacillus Thuringiensis Toxin That Recognizes Cancer Cells. J. Mol. Biol..

[10] Brasseur K., Auger P., Asselin E., Parent S., Cote J.C., Sirois M. (2015). Parasporin-2 from a New Bacillus Thuringiensis 4R2 Strain Induces Caspases Activation and Apoptosis in Human Cancer Cells. PLoS ONE.

[11] Xu C., Wang B.C., Yu Z., Sun M. (2014). Structural Insights into Bacillus Thuringiensis Cry, Cyt and Parasporin Toxins. Toxins.

[12] Periyasamy A., Kkani P., Chandrasekaran B., Ponnusamy S., Viswanathan S., Selvanayagam P., Rajaiah S. (2016). Screening and Characterization of a Non-Insecticidal Bacillus Thuringiensis Strain Producing Parasporal Protein with Selective Toxicity against Human Colon Cancer Cell Lines. Ann. Microbiol..

[13] Kitada S., Abe Y., Maeda T., Shimada H. (2009). Parasporin-2 Requires GPI-Anchored Proteins for the Efficient Cytocidal Action to Human Hepatoma Cells. Toxicology.

[14] Soberon M., Portugal L., Garcia-Gomez B.I., Sanchez J., Onofre J., Gomez I., Pacheco S., Bravo A. (2018). Cell Lines as Models for the Study of Cry Toxins from Bacillus Thuringiensis. Insect Biochem. Mol. Biol..

[15] Suhaili S.H., Karimian H., Stellato M., Lee T.H., Aguilar M.I. (2017). Mitochondrial Outer Membrane Permeabilization: A Focus on the Role of Mitochondrial Membrane Structural Organization. Biophys. Rev..

[16] Suárez-Barrera M.O., Visser L., Pinzón-Reyes E.H., Rondón Villarreal P., Alarcón-Aldana J.S., Rueda-Forero N.J. (2022). Site-Directed Mutants of Parasporin PS2Aa1 with Enhanced Cytotoxic Activity in Colorectal Cancer Cell Lines. Molecules.

[17] Bantel H., Sinha B., Domschke W., Peters G., Schulze-Osthoff K., Jänicke R.U. (2001). Alpha-Toxin Is a Mediator of Staphylococcus Aureus-Induced Cell Death and Activates Caspases via the Intrinsic Death Pathway Independently of Death Receptor Signaling. J. Cell Biol..

[18] Cruz J., Suárez-Barrera M.O., Rondón-Villarreal P., Olarte-Diaz A., Guzmán F., Visser L., Rueda-Forero N.J. (2021). Computational Study, Synthesis and Evaluation of Active Peptides Derived from Parasporin-2 and Spike Protein from Alphacoronavirus against Colorectal Cancer Cells. Biosci. Rep..

[19] Suárez-Barrera M.O., Visser L., Rondón-Villarreal P., Herrera-Pineda D.F., Alarcón-Aldana J.S., Van den Berg A., Orozco J., Pinzón-Reyes E.H., Moreno E., Rueda-Forero N.J. (2021). Genetic Modification Approaches for Parasporins Bacillus Thuringiensis Proteins with Anticancer Activity. Molecules.

[20] Mahalakshmi A., Shenbagarathai R. (2010). Homology Modeling of Cry10Aa Toxin from B. Thuringiensis Israelensis and B. Thuringiensis Subsp. LDC-9. J. Biomol. Struct. Dyn..

[21] Walters F.S., Stacy C.M., Lee M.K., Palekar N., Chen J.S. (2008). An Engineered Chymotrypsin/Cathepsin G Site in Domain I Renders Bacillus Thuringiensis Cry3A Active against Western Corn Rootworm Larvae. Appl. Environ. Microbiol..

[22] Mandal C.C., Gayen S., Basu A., Ghosh K.S., Dasgupta S., Maiti M.K., Sen S.K. (2007). Prediction-Based Protein Engineering of Domain I of Cry2A Entomocidal Toxin of Bacillus Thuringiensis for the Enhancement of Toxicity against Lepidopteran Insects. Protein Eng. Des. Sel..

[23] Gowdhami B., Manojkumar Y., Vimala R.T.V., Ramya V., Karthiyayini B., Kadalmani B., Akbarsha M.A. (2022). Cytotoxic Cobalt (III) Schiff Base Complexes: In Vitro Anti-Proliferative, Oxidative Stress and Gene Expression Studies in Human Breast and Lung Cancer Cells. BioMetals.

[24] Van Opdenbosch N., Lamkanfi M. (2019). Caspases in Cell Death, Inflammation and Disease. Immunity.

[25] Indrayanto G., Putra G.S., Suhud F. (2021). Validation of In-Vitro Bioassay Methods: Application in Herbal Drug Research. Profiles Drug Subst. Excip. Relat. Methodol..

[26] Okumura S., Saitoh H., Ishikawa T., Inouye K., Mizuki E. (2011). Mode of Action of Parasporin-4, a Cytocidal Protein from Bacillus Thuringiensis. Biochim. Biophys. Acta.

[27] Kennedy C.L., Lyras D., Cordner L.M., Melton-Witt J., Emmins J.J., Tweten R.K., Rood J.I. (2009). Pore-Forming Activity of Alpha-Toxin Is Essential for Clostridium Septicum-Mediated Myonecrosis. Infect. Immun..

[28] Pal S., Ray S.D., Homechaudhuri S. (2015). Evaluation of In Vivo Non-Specific Immunity and Oxidative Stress in Labeo Rohita (Hamilton, 1822) Infected with Aeromonas Hydrophila as Biomarker for Early Diagnosis. Int. J. Fish. Aquat. Stud..

[29] Patel B., Kumar P., Banerjee R., Basu M., Pal A., Samanta M., Das S. (2016). Lactobacillus Acidophilus Attenuates Aeromonas Hydrophila Induced Cytotoxicity in Catla Thymus Macrophages by Modulating Oxidative Stress and Inflammation. Mol. Immunol..

[30] Krishnan V., Domanska B., Elhigazi A., Afolabi F., West M.J., Crickmore N. (2017). The Human Cancer Cell Active Toxin Cry41Aa from Bacillus Thuringiensis Acts like Its Insecticidal Counterparts. Biochem. J..

[31] Degiacomi M.T., Iacovache I., Pernot L., Chami M., Kudryashev M., Stahlberg H., van der Goot F.G., Dal Peraro M. (2013). Molecular Assembly of the Aerolysin Pore Reveals a Swirling Membrane-Insertion Mechanism. Nat. Chem. Biol..

[32] Abe Y., Shimada H., Kitada S. (2008). Raft-Targeting and Oligomerization of Parasporin-2, a Bacillus Thuringiensis Crystal Protein with Anti-Tumour Activity. J. Biochem..

[33] Yokosuka T., Goto H., Fujii H., Naruto T., Takeuchi M., Tanoshima R., Kato H., Yanagimachi M., Kajiwara R., Yokota S. (2013). Flow Cytometric Chemosensitivity Assay Using JC-1, a Sensor of Mitochondrial Transmembrane Potential, in Acute Leukemia. Cancer Chemother. Pharmacol..

[34] Kühnel J.M., Perrot J.Y., Faussat A.M., Marie J.P., Schwaller M.A. (1997). Functional Assay of Multidrug Resistant Cells Using JC-1, a Carbocyanine Fluorescent Probe. Leukemia.

[35] Karlsson M., Zhang C., Méar L., Zhong W., Digre A., Katona B., Sjöstedt E., Butler L., Odeberg J., Dusart P. (2021). A Single-Cell Type Transcriptomics Map of Human Tissues. Sci. Adv..

[36] Uhlen M., Zhang C., Lee S., Sjöstedt E., Fagerberg L., Bidkhori G., Benfeitas R., Arif M., Liu Z., Edfors F. (2017). A Pathology Atlas of the Human Cancer Transcriptome. Science.

[37] Berman H.M., Westbrook J., Feng Z., Gilliland G., Bhat T.N., Weissig H., Shindyalov I.N., Bourne P.E. (2000). The Protein Data Bank. Nucleic Acids Res..

[38] Jo S., Kim T., Iyer V.G., Im W. (2008). CHARMM-GUI: A Web-Based Graphical User Interface for CHARMM. J. Comput. Chem..

[39] Klähn M., Zacharias M. (2013). Transformations in Plasma Membranes of Cancerous Cells and Resulting Consequences for Cation Insertion Studied with Molecular Dynamics. Phys. Chem. Chem. Phys..

